# Rapid and real-time monitoring of bacterial growth against antibiotics in solid growth medium using a contactless planar microwave resonator sensor

**DOI:** 10.1038/s41598-021-94139-y

**Published:** 2021-07-20

**Authors:** Mandeep Chhajer Jain, Anupama Vijaya Nadaraja, Rakesh Narang, Mohammad Hossein Zarifi

**Affiliations:** Okanagan Microelectronics and Gigahertz Applications Laboratory, School of Engineering, University of British Columbia, Kelowna, BC V1V 1V7 Canada

**Keywords:** Biomedical engineering, Electrical and electronic engineering

## Abstract

Infection diagnosis and antibiotic susceptibility testing (AST) are pertinent clinical microbiology practices that are in dire need of improvement, due to the inadequacy of current standards in early detection of bacterial response to antibiotics and affordability of contemporarily used methods. This paper presents a novel way to conduct AST which hybridizes disk diffusion AST with microwave resonators for rapid, contactless, and non-invasive sensing and monitoring. In this research, the effect of antibiotic (erythromycin) concentrations on test bacterium, *Escherichia coli* (*E. coli*) cultured on solid agar medium (MH agar) are monitored through employing a microwave split-ring resonator. A one-port microwave resonator operating at a 1.76 GHz resonant frequency, featuring a 5 mm^2^ sensitive sensing region, was designed and optimized to perform this. Upon introducing uninhibited growth of the bacteria, the sensor measured 0.005 dB/hr, with a maximum change of 0.07 dB over the course of 15 hours. The amplitude change decreased to negligible values to signify inhibited growth of the bacteria at higher concentrations of antibiotics, such as a change of 0.005 dB in resonant amplitude variation while using 45 µg of antibiotic. Moreover, this sensor demonstrated decisive results of antibiotic susceptibility in under 6 hours and shows great promise to expand automation to the intricate AST workflow in clinical settings, while providing rapid, sensitive, and non-invasive detection capabilities.

## Introduction

Bacterial infections have become a public health crisis around the world in recent years. Due to the over-prescription of antibiotics and non-compliance with their usage, more types of pathogens have begun to take a route towards further resistance against antibiotics^1–4^. Accordingly, the World Health Organization (WHO) has developed a list of 12 pathogen families which are highly resistant to antibiotics and pose a threat to public health on a global scale^3^. The new found resistance of these pathogens towards antibiotics has created a need for faster and more high-throughput antibiotic susceptibility testing (AST) practices^4,5^.

Current AST methods often suffer from the pitfalls of being extremely expensive, time-consuming, labor-intensive, prone to cross-contamination, and have unstandardized practices^5^. Disk diffusion and broth dilution methods are currently the most widely utilized tests which use various antibiotics in different concentrations against bacterial colonies to test for antibiotic susceptibility or resistance^4–8^. These methods are extremely time-consuming and require upwards of 24 hours of incubation of the bacteria. Furthermore, they display poor performance when analyzing slow-growing bacteria and both methods require expensive resources such as large volumes of reagents and sizeable experimental equipment. Recently, matrix-assisted laser desorption ionization-time of flight mass spectrometry (MALDI-TOF MS) has been deemed efficient in differentiating between various bacteria strains, however, it is far too expensive to operate and maintain for most clinics globally^9,10^. Additionally, newly emerging genotypic methods have been used which utilize polymerase chain reaction (PCR)^11,12^, DNA microarrays^13^, or loop-mediated isothermal amplification (LAMP)^14^, however, their complex and user intensive protocols make them hard to implement in various clinics and situations^4,11^. Other emerging methods of AST which utilize fluorescence^15^, electrochemical^16^, impedimetric^17^, microfluidics^18–22^, microscopy^23^, and bioluminescence^15^ techniques are currently improving the scope of AST. However, they are far too expensive and complex to develop, require laborious protocols to execute, require direct contact with the sample growth medium and need various expensive reagents and equipment, making testing cumbersome^4^. This creates a crucial need for a more efficient and faster method of AST, which is cheap enough to implement and does not require a plethora of resources to operate and maintain.

Microwave sensing technology has recently been adopted in a myriad of applications including ice sensing^24,25^, liquid and gas sensing^26–29^, dielectric spectroscopy^30–32^, monitoring cancer^33,34^, detection of glucose and electrolyte level in aqueous solutions^35,36^, and recently in clinical microbiology practices^37–45^. Microwave resonators have recently shown to be highly sensitive in sensing and monitoring bacteria^39,40,46,47^. Through translating variations of dielectric properties to quantifiable signals such as resonant amplitude and resonant frequency, microwave sensing has shown to be adaptive towards the needs of various biosensing applications and inexpensively implemented without the need to execute laborious protocols^36,39,46,48^. Among the various microwave resonators, planar microwave resonators are easily fabricated, can be reused for multiple assays, and have shown highly sensitive, real-time, and high-throughput results^25,27,49–51^. Microwave resonators have been coupled with microfluidic chips to simulate broth dilution tests and have already displayed high levels of sensitivity in monitoring bacterial growth in various conditions^39,40,52,53^. Furthermore, differential microwave resonators have been implemented to reduce environmental noise in performing a more robust analysis on bioassays^36,46^. However, AST has only been proposed through microwave sensing and not yet demonstrated^39^. This lays on the premise in which antibiotics have proven to impact the growth of microorganisms by targeting protein synthesis, DNA and RNA multiplication, and other metabolic activities to inhibit bacterial growth^54,55^. The change of concentration of charged by-products released during antibiotic-bacterial interaction can indirectly aid in monitoring the metabolic activity through measuring conductivity and permittivity changes resulting in a change of resonant profiles overtime^39,40,47^. Therefore, to tackle the need for faster and more efficient AST practices, microwave sensing can address all of the pitfalls suffered by the current methods employed for AST.

In this study, a simplified model of AST on *Escherichia Coli* (*E. coli*) using microwave split ring-resonators is demonstrated in the absence or presence of various antibiotic concentrations. Among the existing microstrip resonator variations, a frequency variation resonator consisting of a single split ring was employed to monitor and detect the impact of various concentrations of antibiotics on the growth of *E. coli*. Solid MH agar inoculated with *E. coli* is cultured on a microwave resonator ring gap and the transient resonant response of the resonator is dictated by the growth, of the bacterial cells. Upon introducing various concentrations of the antibiotic erythromycin (E), bacteria growth is inhibited which dampens the resonant response of this test, when compared to growing bacteria. This electrical signal is analyzed by a vector network analyzer (VNA) in a real-time, non-invasive, and sensitive manner. Each sample was tested for 15 hours; however, decisive signals of growth inhibition were determined as early as 6 hours. This study not only calibrates a microwave resonator for AST on *E. coli* using erythromycin, but it demonstrates the efficacy of microwave sensors in clinical microbiological practices and has shown to vastly enhance the efficiency of AST.

## Results and discussion

### Electromagnetic field analysis of the sensor

**Figure 1:**
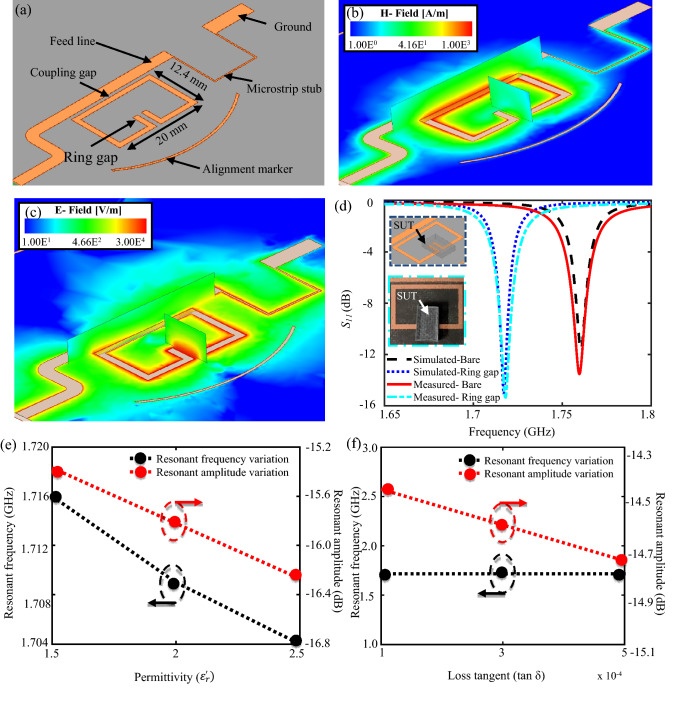
(**a**) Modelled one port split ring resonator in High-Frequency Structure Simulator (HFSS). (**b**) Magnetic field distribution on the resonator. (**c**) Electric field distribution on the resonator with the sensitive zone found in the ring gap. (**d**) Simulated vs measured reflection coefficient of the sensor in the absence and presence of a sample under test (SUT) on the sensitive region of the resonator. Impact of variation in (**e**) permittivity and (**f**) loss tangent of the SUT on the resonant amplitude and resonant frequency of the resonator.

A planar microstrip ring resonator structure was modelled in High-Frequency Structure Simulator (HFSS) to study the operation of the resonator. The resonator was designed to operate at a frequency of 1.76 GHz due to the low effective loss of the solid MH agar media. The resonant structure consists of a half-wavelength split-ring resonator and a feed line, as shown in Fig. 1a. The microstrip resonator was designed on a low loss dielectric substrate (Rogers 5880) with permittivity of 2.2, loss tangent of 0.0009, and thickness of 0.79 mm to minimize losses in the feed line or along the structure. The split ring resonator length was calculated using Eq. (1):^56^.

1 $$l=\frac{c}{2f\sqrt{{\varepsilon }_{r}}}$$

where *l* is the length of the split ring resonator (m), *c* is the speed of light (3 × 10^8^ m/s), *f* is the resonant frequency (1.76 GHz), and ${\varepsilon }_{r}$ is the relative permittivity of the substrate (2.2). Based on Eq. (1), the length of the split ring resonator was calculated to be 57.5 mm. However, the resonant frequency is also governed by the capacitance of the resonant circuit^56^. Since the ring gap of the resonator was modified to monitor an area of 5 mm^2^, the length of the resonator was modified to 73.8 mm to compensate for the increase in the capacitive area and achieve the desired resonant frequency. The coupling gap width was selected as 0.8 mm to achieve maximum magnetic coupling between the feed line and the split ring, as shown in Fig. 1b. The feedline was matched to the electromagnetic (EM) source impedance i.e. 50 Ohm using LineCalc (by Advanced Design Systems 2020), to allow maximum power transfer from the source to the resonator structure. The dimensions of the feed line and the split ring resonator are shown in Fig. 1a.

The resonant structure was designed as a one-port device. One-port devices are widely used for accurately detecting and characterizing conductive dielectric materials^57^. As shown in Fig. 1a, one end of the feedline was connected to an EM source, and the other end was terminated to the ground (through a via extending through the substrate) using a high impedance quarter wavelength microstrip stub. The impedance of the stub was calculated using LineCalc by Advanced Design Systems 2021 and found to be 132.7 Ω. The stub was added to discharge the accumulated charge between the plastic plate sample container and the feed line due to the presence of weak capacitances between them. The high impedance of the stub prevented loading of the matched feed line while simultaneously acting as an open circuit to generate a microwave signal at the resonance frequency and short circuit at DC frequencies. An alignment marker was added to the design to align the inoculated bacterial culture with the sensitive region of the resonator. Since the most sensitive area of the resonator lies around its ring gap, bacterial growth must occur close to the ring gap to achieve successful bacterial growth monitoring. The simulated response of the bare sensor in the absence of test materials is presented in Fig. 1d, shown in black, in which the resonant frequency and amplitude were found to be 1.76 GHz and − 11.58 dB, respectively.

To determine the sensitive region of the resonator, full 3D electromagnetic field simulations were performed in HFSS. The sensitive regions of the resonator are the regions displaying the maximum magnitude of the electric field (E-field) shown in red within Fig. 1c. This figure presents the E-field distribution on the microstrip resonator sensor at the operating resonant frequency of 1.76 GHz. The electric field density reached a maximum magnitude around the ring gap as compared to other regions of the sensor and was thus selected as the sensitive region of the resonator. Additionally, to validate the determined sensitive region, a system under test (SUT) was placed on the ring gap, and the simulated response was analyzed as shown in Fig. 1d. For the SUT in this instance, a standard test material with a permittivity of 2.2, loss tangent of 0.0009, and physical dimensions of 6 × 9 × 1.57 mm was used. In effect, the overall effective dielectric properties at the sensitive region were modified, resulting in a shift of the resonant profile of the resonator. The material was placed at a distance of 0.1 mm above the ring gap to further validate contactless sensing as a viable application for microwave-based sensing for AST. Based on the simulation results, a − 3.42 dB and 44 MHz shift in the resonant amplitude and resonant frequency, respectively, was observed, indicating that the ring gap was the most sensitive to the presence of material and was chosen for further analysis.

In another study, an investigation to understand the impact of variation in the dielectric properties of the SUT on the response of the resonator was undertaken, and the results are presented in Fig. 1e,f. The permittivity and loss tangent of the SUT were varied independently and the other physical properties of the material were held constant. Based on the simulation results, resonant amplitude and resonant frequency were found to be inversely proportional to the variation in the permittivity of the SUT. However, variation in the loss tangent or conductivity only impacted the resonant amplitude of the sensor whereas, the resonant frequency remained unchanged. Since bacterial growth significantly impacts the conductivity of the growth medium, resonant amplitude variations were selected as a dominant parameter to study the variation in the loss tangent or conductivity of the SUT.

Finally, the measured reflection gain of the fabricated resonator was measured and compared against the simulated results. In the absence of any test material, the resonant frequency and resonant amplitude of the fabricated sensor was found to be 1.76 GHz and − 13.5 dB, respectively, resulting in a 0.05% and 16.75% error between the simulated and measured values for the resonant frequency and amplitude, respectively, which closely follows the simulation results shown in Fig. 1d. The deviation in the resonant amplitude between the simulated and measured values can be attributed to imperfections in the fabrication process, which includes soldering of SMA connectors, and the placement of the via for a ground. Furthermore, the sensitive region of the fabricated resonator was verified by placing a standard test sample (Rogers RT/duroid 5880) with a relative permittivity (${\varepsilon }_{r })$ of 2.2, loss tangent (tan δ) of 0.0009, and dimensions of 6 × 9 × 1.79 mm on the ring gap of the resonator. A distinct shift in the resonant amplitude and resonant frequency by − 1.48 dB and 42 MHz, respectively, was observed when the material was placed on the ring gap, resulting in a 0.05% and 1.925% error between the experimental and theoretical values of the resonant amplitude and frequency, respectively, which agrees with the simulation results shown in Fig. 1d. This confirmed the sensitivity of the selected region to the presence of test material and was therefore employed to monitor the impact of antibiotics on the growth of *E. coli*.

### Initial antibiotic susceptibility disk diffusion calibration discussion

**Figure 2:**
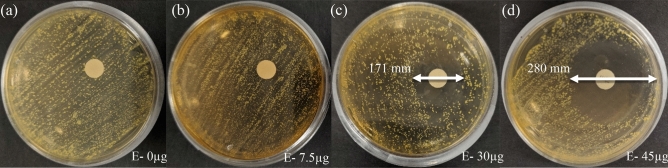
Captured images revealing the impact of **(a)** 0, **(b)** 7.5, **(c)** 30, and **(d)** 45 µg of erythromycin on the growth of *E. coli*.

Prior to utilizing the microwave resonator to monitor the impact of antibiotics on the growth of microorganisms, a preliminary disk diffusion test was performed. In this test, the growth of *E. coli* was visually observed against various concentrations of erythromycin. Based on the captured images presented in Fig. 2, different microbial responses were observed at various concentrations of erythromycin. Low concentrations of erythromycin (E-0 and E-7.5) did not inhibit microbial growth as there were no visible zones of inhibited growth. However, E-30 and E-45 resulted in the formation of zones of inhibited growth (171 mm and 280 mm respectively). This experiment verifies that *E. coli* HB101 is susceptible to E-30 and E-45, as used in this study. However, it should be noted that the bacteria are more susceptible to E-45 than E-30.

### Microwave and image-based detection of bacterial growth against antibiotics

**Figure 3:**
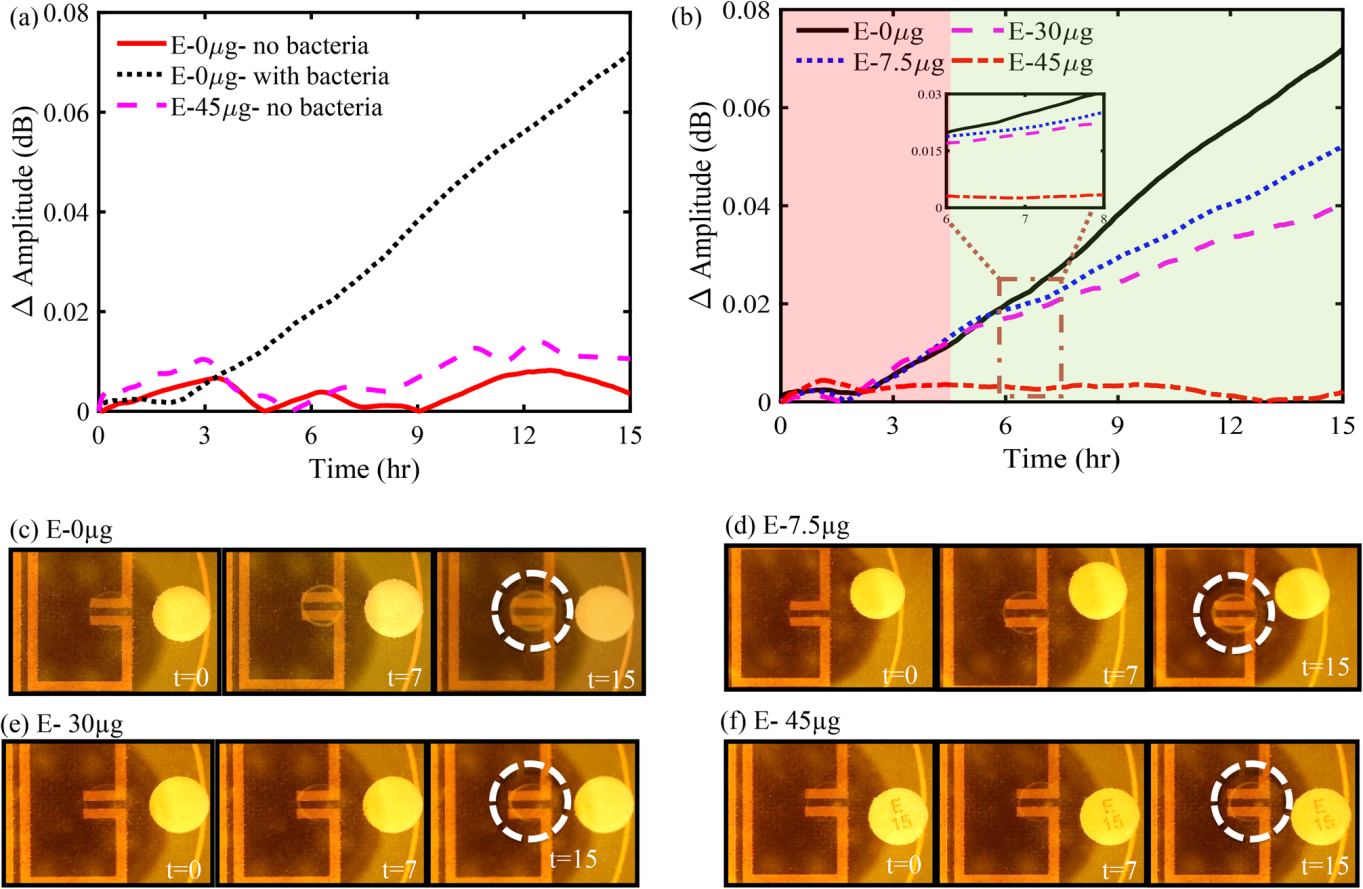
(**a**) Impact of a blank paper disk (without antibiotics) with inoculated and un-inoculated agar samples, and a 45 µg erythromycin disk with inoculated agar sample on the response of the resonator. (**b**) ΔAmplitude (dB) vs time (hr) depicting the detectable (green) and undetectable (red) regions of bacterial growth, (**c**–**f**) microscope images, of 0, 7.5, 30, and 45 µg of erythromycin with bacterial growth or inhibition shown in the dotted white circle.

A study to determine the impact of a hydrated blank paper disk (without antibiotics) and a 45 µg erythromycin disk on the response of the resonator was performed to establish a baseline response to distinguish between detectable and undetectable regions of microbial growth (Fig. 3a). It is evident from the measured results that the presence of a blank disk on a solid agar plate (with no bacteria) had a negligible impact on the response of the resonator as opposed to the ΔAmplitude (dB) measured for an inoculated solid agar plate with a blank disk (E-0 µg). The impact of an E-45 disk placed on a solid agar plate (with no bacteria) on the response of the resonator is also presented in Fig. 3a. The measured resonant amplitude variation was insignificant in comparison to the measured resonant amplitude variation for a solid agar plate inoculated with bacteria and accompanied with a blank disk (E-0 µg). Hence, the maximum variation in the resonant amplitude, i.e., 0.015 dB, was considered as a threshold to determine the regions of undetectable and detectable bacterial growth.

Figure 3b presents the variation in the resonant amplitude corresponding to bacterial growth at tested antibiotic concentrations (0, 7.5, 30, and 45 µg). The figure shows a clear distinction between the measured *E. coli* growth at various concentrations of erythromycin. During bacterial growth, bacteria consume the nutrients readily available in their vicinity while synthesizing proteins and several charged byproducts^55,58–62^. The charged byproducts expelled into the surrounding media leads to conductivity changes in the growth medium, which is reflected in the resonant amplitude variation of the microstrip resonator sensor.

**Figure 4:**
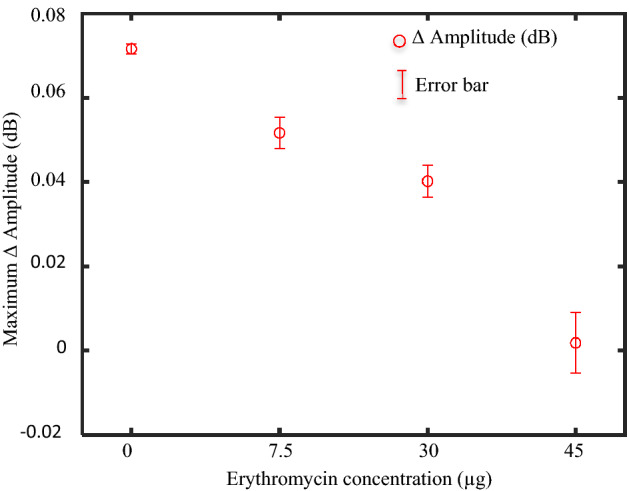
Maximum resonant amplitude variation (Δ Amplitude (dB)) for various concentrations of erythromycin and their corresponding mean and standard deviation.

In the absence of erythromycin, a maximum resonant amplitude variation of 0.07 dB was observed for the control plate (E- 0 µg) due to unrestricted bacterial growth around the sensitive region of the sensor (Fig. 3c). However, in the presence of erythromycin, the protein synthesis during bacterial metabolism was hindered, thereby impacting bacterial growth. As evident from Fig. 3b, the rate of change of resonant amplitude, which can be associated with the growth rate of bacteria, decreased as the concentration of erythromycin increased, which is in positive correlation with the microscopic images captured at constant time intervals (Fig. 3c–f). The slope of the measured resonant amplitude variation in the detectable bacterial growth region was calculated and found to be 0.005, 0.003, 0.002 dB/hr. with a coefficient of determination (R^2^) of 0.999, 0.998, and 0.997 for 0, 7.5, and 30 µg of erythromycin, respectively. Furthermore, an insignificant change in the ΔAmplitude (dB) of 0.005 dB at 45 µg erythromycin concentration indicated a complete inhibition of bacteria growth and was supported by microscopic images captured at constant time intervals (Fig. 3f). All the experiments were repeated thrice and the average of maximum ΔAmplitude (dB) vs erythromycin concentration (µg) with their corresponding uncertainties in the measurement are presented in Fig. 4 and Supplementary Table ST1.

Bacterial susceptibility to antibiotics has shown to be concentration-dependent^63,64^. Low doses of erythromycin decrease protein synthesis in bacteria, thereby reducing the growth rate rather than inhibiting bacterial growth completely. This is in accordance with the bacteriostatic nature of erythromycin. However, higher concentrations of erythromycin completely inhibited bacteria growth leading to the formation of zones of inhibition as shown in Fig. 2 in the previous experiement^63–65^. It is evident from the figure that a high concentration of erythromycin leads to a large zone of inhibition, and vice versa. Correlating these results with Fig. 3 explains not only the greater zone of inhibition for E-45 compared to E-30 and E-7.5 but also the difference of rates of change in the differential resonant amplitude over the course of the 15 hours measured by the microwave resonator. A noticeable difference can be seen in Fig. 3b when comparing the differential responses of E-30 and E-7.5. Although subtle, E-30 showed a more dampened response in resonant amplitude change over time and had a lesser overall change than E-7.5, which is attributed to the reduced growth or metabolic activity of the bacteria. Therefore, it can be concluded that as the concentration of the erythromycin increased, the diameter of the zone of inhibition increased, overlapping the sensitive region of the microwave sensor. Consequently, the bacteria growth in the sensitive region decreased, which is in positive correlation with the measured microwave sensor’s response and is supported by microscope images captured at constant time intervals (Fig. 3c–f). Thus, it can be concluded that placing the antibiotic disks closer to the resonator will also impact the time at which decisive AST can be conclusive. However, the distance between the antibiotic disk and the resonator gap can be optimized as low concentrations marginally inhibiting growth can lead to inaccuracies for susceptibility in different concentrations.

A noteworthy outcome of this work was the sensor’s ability to successfully distinguish the impact of different concentrations of erythromycin on the growth of *E. coli* before any visible cues. The impact of various concentrations of antibiotics on *E. coli* only became apparent through visual inspection at T = 15 hr with no distinct or observable impact in the initial hours of the study. However, the microwave sensor was able to clearly distinguish the impact of erythromycin on *E. coli* growth within the first 6 hours of the experiment, which is far lower than the technical capacity of conventional microbiological disk diffusion studies. The strength of the proposed sensing technique in contrast to so some of the state-of-the-art biosensors for AST is presented in Table 1. Therefore, the rapid, contactless, non-invasive, portable, inexpensive, and reusable nature of the planar microwave resonator makes it a promising tool in the field of microbiology.

**Table 1:** Comparison of the state-of-the-art biosensors for antibiotic susceptibility testing.

Sensor type/technique	Time taken for AST	Potential limitations		Reference
Electrochemical biosensor	60 min	Sensor is in direct contact with the growth medium, complex and expensive fabrication process		^66^
Impedimetric biosensor	1–2 h	Sensor is in direct contact with the growth medium		^17^
Kirby Bauer AST Technique	24- 48 h	Labor intensive, time-consuming		^7^
Bio-electrodes	4 h	Electrodes need to be in direct contact with the growth medium and limit reusability		^67^
Microwave resonator sensor	6 h	Alignment of antibiotic can impact the time to affect the inoculated bacterial culture		This work

In summary, a planar microwave resonator sensor was implemented to monitor the impact of antibiotics on the growth of microorganisms. The proposed sensing platform offers the early detection of microbial growth by monitoring the conductivity variations of the growth medium due to microbial growth under the influence of antibiotics. Early detection of the response of microbial growth to antibiotics can be beneficial in developing treatment strategies against infections. The positive correlation between the measured microwave response of *E. coli* growth against the tested erythromycin concentrations along with the microscopic images and the preliminary analysis of zones of inhibition successfully demonstrated the efficacy of the proposed system. This work has shown promise for further automation of AST and increasing efficiencies within clinical microbiology labs for faster and more robust results for the proper care of bacterial infections.

## Methods and materials

### Reagents and materials

HB101 *E.coli* was used as the test microorganism, which was cultured in Muller Hinton agar (C6421) (MH Agar) as the growth media was purchased from Hardy Diagnostics CRITERION. BD BBL 15 µg Erythromycin paper disk, BD BBL 6 mm Blank paper disk, and MilliporeSigma Erythromycin (powdered form) were procured from Fisher Scientific, Canada. Ammonium persulfate as an etchant to fabricate a microstrip sensor was purchased from MG Chemicals (Surrey, Canada). Dielectric laminates were provided by Rogers Corp. USA. A Rohde and Schwarz ZNB20 VNA was used for the sensor measurement.

### Bacterial samples preparation

Mueller Hinton Agar (MH agar) was used to prepare growth media for this experiment. 1.91 mg of MH agar was thoroughly mixed with 50 mL of deionized water and sterilized for 15 minutes at 121 °C using an autoclave. The media was cooled down to 50 °C, and 3 mL of the sterilized media was poured into a Petri dish to achieve an agar thickness of 1 mm. The plates were cooled down to room temperature, and 3 µL of *E. coli* at OD_600_ = 1.5 were inoculated at the predesignated position.

The antibiotic disks were prepared by soaking a blank paper disk with 30 µL of the desired erythromycin concentration. The disk was transferred to the inoculated plate and placed at a constant distance of 10 mm from the inoculated *E. coli*. The Petri dish was sealed using a parafilm to avoid changes in the Petri dish due to external ambient factors.

### Fabrication of microwave resonator

A microstrip planar ring resonator sensor was fabricated on a Rogers RO5880 laminate from Rogers Corporation Pvt. Ltd. with a dielectric thickness of 0.79 mm, copper cladding thickness of 35 µm, permittivity of 2.2, and loss tangent of 0.0009. The resonator pattern was etched following previously used protocols^53^. Briefly, the split ring design was designed and simulated in ANSYS HFSS to optimize parameters and operating resonant profiles. Subsequently, the layout of the resonator was patterned using a laminator by incorporating several heating and pressing cycles. The patterned laminate was etched using ammonium persulfate. One end of the feedline was connected to a soldered SMA connector, and the other end was grounded. The modelled resonator structure and the simulation and fabrication results are discussed in detail in the above sections.

### Initial antibiotic susceptibility disk diffusion calibration

To demonstrate the impact of 0, 7.5, 30, and 45 µL of erythromycin on the growth of *E. coli*, MH agar plates were inoculated using the streak plate method. Blank paper disks were soaked in 30 µL of RO water with 7.5, 30, and 45 µg of erythromycin, and tapped on the surface of the growth medium. The petri dish was sealed using parafilm to avoid precipitation and fungal contamination. The petri dish was incubated at 22 °C for 36 h and the diameters of the zone of inhibition were measured. The captured visual microbial growth at the end of this study is discussed in detail in the above sections.

### Experimental setup of the sensor and VNA

**Figure 5:**
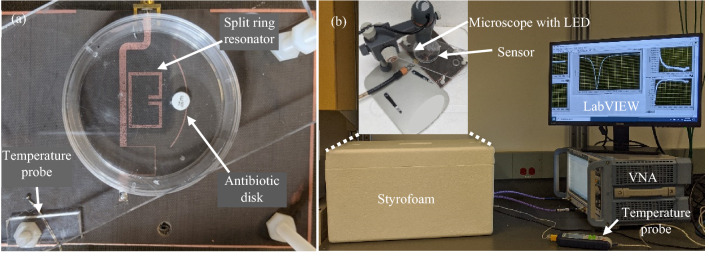
Fabricated sensor arrangement and the experimental setup for measurement of bacterial growth against antibiotics. (**a**) The fabricated planar microstrip resonator with sample petri dish placed strategically on the resonator. (**b**) Experimental setup consisting of a thermally insulated styrofoam enclosure equipped with the sensor, temperature probe, microscope with LED, and vector network analyzer (VNA).

The fabricated sensor arrangement shown in Fig. 5a was enclosed in a thermally insulated and mechanically stabilized styrofoam enclosure equipped with a LED light source, a digital microscope, and a temperature probe. The temperature probe was employed to monitor the variations in the ambient temperature. The ambient temperature was recorded to be 23.25 ± 0.25 °C. The bacterial growth images were captured at fixed time intervals using a 10 × Celestron digital microscope. The experimental setup is demonstrated in Fig. 5b.

The inoculated plate was strategically positioned (Fig. 5a) and firmly secured on the sensor such that the distance between the paper disk and the ring gap was 1 cm. The sensor was connected to a ZNB20 Vector Network Analyzer (VNA) by Rohde and Schwarz. The VNA was calibrated every 15 hours to minimize the impact of drift in the measurement instrument on the response of the resonator. The VNA was operated between 1.55 GHz to 1.8 GHz. The intermediate frequency (IF) bandwidth was set to 300 Hz to minimize the broadband noise, and the number of points was selected as 6401 to increase the sampling resolution. The output power of the VNA was set to 0 dBm i.e. 1 mW which was sufficiently low to have no impact on the growth of *E. coli* through adverse effects just as Joule heating^68,69^. The VNA was triggered to measure the reflection coefficient (*S*_11_ dB) every two minutes using an automated LabVIEW program made in-house.

To test the ability of the sensor to monitor the susceptibility of *E.coli* to various concentrations of the antibiotic, erythromycin (7.5, 30, and 45 µg), the reflection gain (*S*_11_ (dB)) of the sensor was measured and recorded using VNA and LabVIEW, respectively. The measured *S*_11_ (dB) was normalized by subtracting from the initially measured value due to day-to-day variations of ambient conditions. Furthermore, the data pertaining to the initial 90 minutes were excluded to account for the stabilization of the measurement apparatus.

## Supplementary Information

Supplementary Information.
