# Supplementary material for: Rapid and real-time monitoring of bacterial growth against antibiotics in solid growth medium using a contactless planar microwave resonator sensor
Source: Sci Rep. 2021 Jul 20;11:14775. doi: 10.1038/s41598-021-94139-y (PMC8292355; doi:10.1038/s41598-021-94139-y)
Supplement: Supplementary file 1 — Supplementary Information. [file 41598_2021_94139_MOESM1_ESM.docx]

**Supplementary Information**

**Rapid and Real-time Monitoring of Bacterial Growth Against Antibiotics in Solid Growth Medium Using a Contactless Planar Microwave Resonator Sensor**

Mandeep Chhajer Jain^1^, Anupama Vijaya Nadaraja^1^, Rakesh Narang^1^, Mohammad Hossein Zarifi^1*^

*^1^Okanagan Microelectronics and Gigahertz Applicassstions Laboratory, School of Engineering, University of British Columbia, Kelowna, BC V1V 1V7, Canada*

Corresponding Authors:

Mohammad Hossein Zarifi, School of Engineering, The University of British Columbia, Kelowna, BC, V1V 1V7, Canada, Email: [mohammad.zarifi@ubc.ca](mailto:mohammad.zarifi@ubc.ca)

**Table ST1**: Calculated mean and standard deviations for the measured Δ amplitude (dB) at various concentrations of antibiotics.

| Erythromycin Concentration (µg) | Mean (dB) | Standard deviation (dB) |
| --- | --- | --- |
| 0 | 0.053989 | 0.001153 |
| 7.5 | 0.036349 | 0.003755 |
| 30 | 0.021017 | 0.003825 |
| 45 | 0.001824 | 0.007196 |
